# High-quality and field resilient microwave resonators on Ge quantum well heterostructures

**DOI:** 10.1038/s41534-026-01297-w

**Published:** 2026-06-11

**Authors:** Luigi Ruggiero, Carlo Ciaccia, Pauline Drexler, Vera Jo Weibel, Christian Olsen, Christian Schönenberger, Dominique Bougeard, Andrea Hofmann

**Affiliations:** 1 Department of Physics, University of Basel, Basel, Switzerland; 2 University of Regensburg, Regensburg, Germany; 3 Swiss Nanoscience Institute, Basel, Switzerland; 4 YQuantum, Villigen, Switzerland

**Keywords:** Materials science, Nanoscience and technology, Physics

## Abstract

Superconducting resonators integrated with germanium (Ge) quantum wells (QWs) offer a promising platform for hybrid quantum devices. Yet, in the most common heterostructure architectures, they have so far been limited by sizable photon losses. Here, we report the fabrication and characterization of microwave resonators patterned in the aluminum (Al) thin film of an in-situ grown superconductor/semiconductor hybrid heterostructure (HS). The semiconductor part of this hybrid HS is grown on a commercial Ge substrate. We consistently achieve internal quality factors *Q*_*i*_ > 1000, surpassing previous results on Ge QW heterostructures grown using the concept of a virtual Ge substrate on silicon (Si) substrates. We reach *Q*_*i*_ ≈ 49,000 at single-photon occupation and a plateau of *Q*_*i*_ ≈ 20,000 at sub-one photon, an order of magnitude larger than any previously reported value of resonators on Ge QW structures at low power. We further characterize the thin Al film forming the resonator, extracting its kinetic inductance and superconducting gap, and studying its magnetic field dependence. Notably, the resonance remains well-defined up to in-plane magnetic fields of 850 mT. A hysteresis emerges in the out-of-plane magnetic field dependence, for both the resonance frequency and the quality factor, indicating an interesting interplay between vortex-and quasiparticle loss mechanisms.

## Introduction

Strained Ge QWs are a promising platform for both both spin qubits^1–3^ and hybrid quantum devices^4–6^. The platform profits from the absence of piezoelectricity and hyperfine interaction inherent to group-IV materials while also enabling a superconducting proximity effect. Integration of these devices with microwave resonators^7–11^ is attractive as it enables fast, high-fidelity readout and offers a route toward long-range qubit coupling via cavity photons^12–14^. In planar Ge/SiGe HS, combining a compact heterostructure architecture with high-quality coplanar waveguide resonators (CPWs) has been challenging so far, mainly due to the lossy substrate originating from specific growth techniques. The strained Ge QW is built by growing a thin layer of Ge between two strain-relaxed Ge-rich SiGe layers. Starting from a commercial Si substrate, the unstrained SiGe layer is obtained via a strain-relaxing buffer^15^, either by gradually increasing the Ge content, or by first growing a pure Ge layer and subsequent decreasing of the Ge content. This latter approach has recently gained increased attention due to the reported high mobilities and successful qubit operations performed on such heterostructures^2,16^. However, it contains many dislocations at the Si-Ge interface, due to the large lattice mismatch of the two materials. The microwave losses introduced by these defects limit the reported *Q*_*i*_ to about 1000^11^. On the other hand, larger *Q*_*i*_ values of a few thousand have been achieved^7–10^ by the former approach at the cost of thick buffer layers and long growth times. These larger quality factors are achieved with both ex-situ molecular-beam-epitaxy (MBE)-grown^17^ or thermally evaporated^10^ Al films. A novel approach has demonstrated promising results of CPWs placed directly on the Si substrate^18^ which is reached using deep etching techniques. However, this requires additional non-trivial fabrication steps, and the effect of the steep etching profile on the quality of the CPW has not been discussed. Also, flip-chip based techniques have helped mitigating the microwave losses^19,20^, at the cost of increased complexity. Additionally, most studies on CPWs on Ge have so far been limited to low magnetic fields, even though analyses on field resilience^8^ are crucial for qubit applications. High quality and high resilient CPWs are crucial ingredients for exploring potential topological superconductivity in hybrid devices^21^ or study the effect of high in-plane fields on the Andreev-Bound-State (ABS) spectrum^22^.

Here, we present an approach delivering high *Q*_*i*_ and a large in-plane magnetic field resilience for CPWs which are structured into a MBE grown superconductor/semiconductor hybrid HS. Different from many existing Ge/SiGe HSs^1–11^, our hybrid HS is grown on a commercial Ge substrate with appropriate grading to a constant SiGe composition^23^. This allows us to avoid the dislocation density-rich Si-Ge interface, which provides loss-channels for the CPW^7^, and still keep the HS stack compact. The hybrid HS is furthermore characterized by an in-situ grown thin superconducting Al film, brought onto the semiconductor without breaking the vacuum. To characterize the material platform and its suitability for microwave technology, we systematically compare eight CPWs of varying lengths and investigate the dependence of *Q*_*i*_ and resonance frequency on applied magnetic fields. Our approach yields CPWs with quality factors compatible with gatemon qubit experiments^9,10^ and which are still operational at a Zeeman energy of around 10 μeV.

## Results

### Resonator characterization

**Fig. 1:**
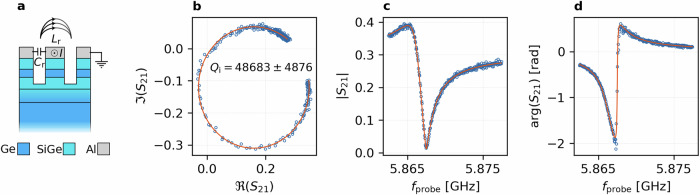
High-Q CPW on a Ge QW. **a** Cross-sectional schematic (not to scale) of a microwave CPW patterned from an Al film deposited on a Ge/SiGe quantum well, which is epitaxially grown on a Ge substrate. **b**–**d** Fit (orange solid line) of the I/Q, transmission amplitude and phase response, respectively. The extracted quality factor exceeds 4.8 × 10^4^ at an average circulating photon number of < *n*_*p*_ > ≃ 1.0.

The MBE-grown hybrid superconductor/semiconductor HS used here features a nominally 8 nm thin Al film directly grown onto a Ge/SiGe HS with a Ge QW. The whole stack, including the 475 μm thick commercial Ge substrate, is shown in Fig. 1(a).

We measure three samples: Sample A with eight *λ*/4 CPWs hanging to a transmission line (TL), and samples B and C with a single *λ*/4 CPW hanging to a TL. In all the samples, the TL is much longer than the CPWs and designed to be 50*Ω*-matched at both ends (see Supplementary Note 1) in order to avoid interference of standing waves. The experiment is designed such that we can separately estimate the internal quality factor *Q*_*i*_ and the coupling quality factor *Q*_*c*_, from which we extract the loaded quality from 1/*Q*_*l*_ = 1/*Q*_*i*_ + 1/*Q*_*c*_.

In a first set of experiments, sample A is cooled down in the dilution refrigerator (DR) with a passive Al magnetic field shield. The ground plane is connected through several Al bonds to the ground of the cryostat. Additional bonds bridging across each CPW and the TL aim to further reduce potential inhomogeneities of the ground plane. The Printed Circuit Board (PCB) is embedded in a copper (Cu) box in order to minimize the impact of standing waves.

With this setup, we achieve an internal quality factor *Q*_*i*_ = (48,683 ± 4876) at low input power *P*_*in*_ corresponding to an average number of photons $< {n}_{p} > =(2{P}_{in}{Q}_{l}^{2})(\hslash \pi {f}_{r}{Q}_{c})\simeq 1$ circulating in the CPW^24^. The internal *Q*_*i*_ and external quality factor *Q*_*c*_, as well as the CPW resonance frequency, are extracted from a circular fit^25^ of the CPW response in the complex plane, as shown in Fig. 1(b - d). The resonance frequency, *f*_*r*_, has been determined by measuring the complex transmission as a function of frequency, where the absolute value shows a dip and the phase response shows a characteristic jump at resonance, see Fig. 1(c) and (d), respectively. The extracted *f*_*r*_ amounts to 5.86 GHz and the coupling quality factor is *Q*_*c*_ = (2531 ± 21). Although here, the coupling quality factor limits the photon lifetime, the design can easily be adjusted to a higher *Q*_*c*_. Meanwhile, the extracted *Q*_*i*_ corresponds to a photon lifetime of *Q*_*i*_/(2*πf*_*r*_) ≃ 2.6 *μ*s, which exceeds the coherence times of many reported qubits in Ge^1,2,26^.

### Film characterization

In contrast to other experiments performed on Ge/SiGe HSs where ex-situ deposited Al layers are used to induce a proximity effect^17^, our CPWs are directly fabricated from a hybrid HS (see Supplementary Fig. 1). The MBE-approach allows to form an abrupt superconductor/semiconductor interface (see transmission electron microscopy (TEM) in Supplementary Fig. 1), via crystalline growth in an ultra-high vacuum (UHV) environment.

**Fig. 2:**
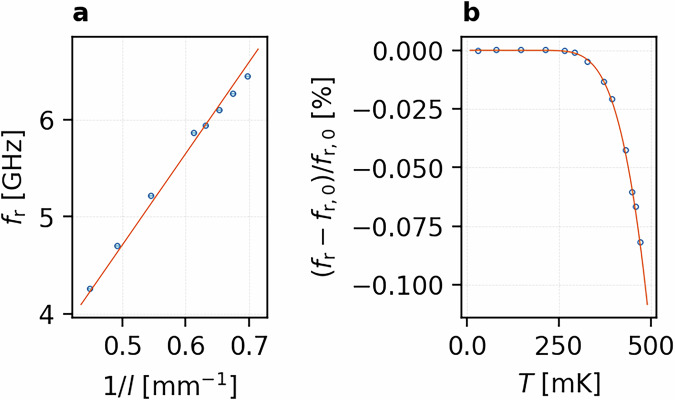
Thin film properties extracted from CPW characteristics. **a** The resonance frequency *f*_*r*_ is inversely proportional to the CPW length *l*. The data (blue dots) are fitted (solid line) to the equation *f*_*r*_(*l*) specified in the main text, yielding a kinetic inductance of (36 ± 2) pH/□ **b** The relative shift of the resonance frequency *f*_*r*_ (blue dots) measured at elevated temperature compared to *f*_*r*,0_ ≔ *f*_*r*,*T*=10 mK_. The solid line is a fit to the BCS theory (see main text), which predicts a decreasing resonance frequency due to the lower density of Cooper pairs, which increases the kinetic inductance.

We first characterize the kinetic inductance *L*_*kin*_ of the Al film. To do so, we measure the dependence of the resonance frequency on the CPW’s length *l*, as shown in Fig. 2(a). We fit our data to ${f}_{r}(l)=1/4l\sqrt{{L}_{{\rm{r}}}{C}_{{\rm{r}}}}$ valid for the employed quarter-wavelength geometry. Here, *L*_r_ = *L*_geo_ + *L*_kin_, ${L}_{geo}=({\mu }_{0}/4)(K({k}_{0}^{{\prime} })/K({k}_{0}))=26.6\,{\rm{nH}}/{\rm{m}}$ is the geometric inductance per unit length, ${C}_{{\rm{r}}}=4{\epsilon }_{0}{\epsilon }_{{\rm{eff}}}K({k}_{0}^{{\prime} })/K({k}_{0})$ is the capacitance per unit length^27^, and *ϵ*_eff_ the material’s dielectric constant. Using the dielectric constant and kinetic inductance as fitting parameters, we extract *ε*_*eff*_ = (10.01 ± 0.01) and *L*_*kin*_ = (35 ± 2) pH/□. We find that the kinetic inductance is four times larger than reported for a 10 nm thick Al film grown ex-situ onto a QW HS in ultra-high vacuum environment^17^. While an increased *L*_*kin*_ is consistent with a thinner superconducting film, the increase by a factor of four suggests that the film might actually be even thinner than the nominal 8 nm.

To further characterize the Al film in terms of its superconducting properties, we analyze the resonance frequency as a function of temperature. It follows a Bardeen-Cooper-Schrieffer (BCS)–like behavior, where the Cooper pair density decreases at elevated temperature, increasing the kinetic inductance and reducing the resonance frequency. We fit $({f}_{r}-{f}_{r,10{\rm{mK}}})/{f}_{r,10{\rm{mK}}}=-\alpha /2\sqrt{\pi {\Delta }_{0}/(2{k}_{B}T)}\exp (-{\Delta }_{0}/({k}_{B}T))$ to the data shown in Fig. 2(b)^28,29^, where *f*_*r*,10 mK_ refers to the resonance frequency at 10 mK and *α* = *L*_*kin*,0_/(*L*_*kin*,0_ + *L*_*geo*_) is the kinetic inductance fraction at zero temperature. We extract a superconducting gap at zero temperature *Δ*_0_ = (298 ± 4) *μ*eV, slightly higher than what has been reported for an ex-situ grown 10 nm thick Al film^17^. Further, we extract the kinetic inductance fraction *α* = (0.75 ± 0.08) from the fit, in agreement with the value obtained from calculating *α*_*sim*_ = 0.85 from the inductance values *L*_*kin*_ and *L*_*geo*_. Together with the increased kinetic inductance, the enhanced *Δ*_0_ is a further indication that our Al film is thinner than the nominal 8 nm^30^.

### Statistics on *Q*_*i*_

**Fig. 3:**
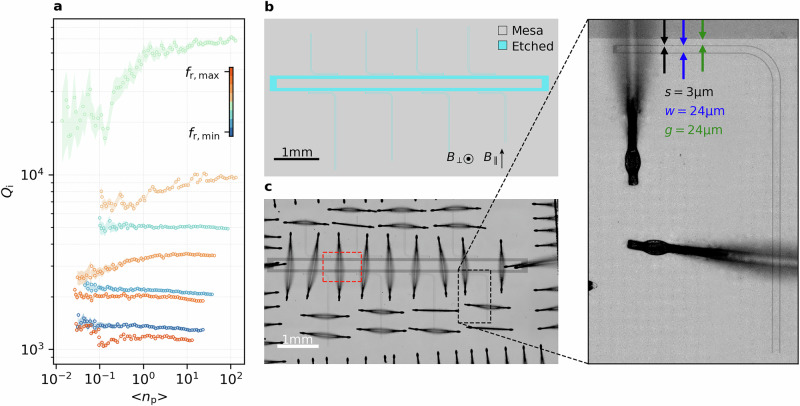
Photon number dependence of eight *λ*/4 CPWs connected to a single TL. **a** Quality factor as a function of average photon number for eight CPWs of different frequencies ranging from about 4 GHz to 7 GHz. **b** Design scheme with eight *λ*/4 CPWs of different length *l* hanging to a common TL. **c** Top view of the sample on which the design in (**b**) is patterned. The impedance of the CPWs at resonance frequency is ${Z}_{r}=\sqrt{{L}_{r}/{C}_{r}}\,\simeq \,66\Omega$^27^. The constant coupling length of 473 μm (not shown) and its relative variation to CPW length yields a variable coupling quality factor *Q*_*c*_ ranging from 1200 to 9000 in agreement with the simulations shown in SFig.2(b). It is also affected by grounding inhomogeneities, which we try to minimize by bonds connecting different areas of the ground plane. The area marked by red dashed line indicates a region of inhomogeneous etching. (c.inset) Zoom-in on the CPW with the highest quality factor, with details on the feedline width *w* = 24 μm, gap to ground *s* = 3 μm, coupling *g* = 24 μm, constant for all eight CPWs.

To gain insight into the variability of our CPWs, we analyze and compare the performance of eight different CPWs all patterned on the same sample A. We provide further information on chip-to-chip variability in the Supplementary Fig. 4. In Fig. 3(a) we plot *Q*_*i*_ for all eight CPWs as a function of average photon number. A sketch of the design and an optical image of the bonded device are shown in Fig. 3 (b) and (c), respectively. The inset of Fig. 3(c) highlights the position of the best CPW relative to the whole sample and shows all relevant dimensions. The chosen design yields impedance values slightly above 50*Ω*, and we consistently measure quality factors exceeding 1000. Thus, higher than what previously reported for CPWs on Ge/SiGe HSs grown via Ge virtual substrates on commercial Si substrates^11^ and similar to those on HSs grown directly on commercial Si^9,17^. This corroborates previous suspicions^11^ that the defects at the interface between the Si substrate and the Ge virtual substrate are the main source of loss.

The origin of the losses can be better understood by sweeping the input power circulating in the CPW. The *Q*_*i*_ of some of the CPWs exhibits an increase with power before saturating at higher levels. This behavior is characteristic of CPW loss dominated by two-level systems (TLS), which cause dissipation at low photon numbers^31^. As the power increases, the TLS gradually saturates, reducing their contribution to loss and resulting in a plateau in *Q*_*i*_ at high power. The onset of this increase depends on factors such as the resonance frequency, the coupling strength to TLS, and the internal quality factor at low photon numbers. For most of our CPWs, we observe this increase in *Q*_*i*_ already at relatively low powers. Notably, in the CPW with the highest observed *Q*_*i*_, we also detect a plateau at low photon number, indicating a regime where unsaturated TLS dominate the loss^31^. In this case, *Q*_*i*_ plateaus at ~20,000 at average photon numbers well below one.

For some of the CPWs, we observe no pronounced dependence of *Q*_*i*_ on input power, suggesting that loss mechanisms other than TLS dominate^11^. One possible source is an etching imperfection in the TL, as indicated by a dashed red rectangle in Fig. 3(c), which may disrupt the homogeneity of ground currents or promote quasiparticle excitations. The relative significance of TLS loss—quantified by the difference in *Q*_*i*_ between low and high power or *Q*_*i*,max_—clearly correlates with the CPW’s proximity to this defect (see Supplementary Fig. 2). Additionally, the placement of bond wires has been shown to influence CPW performance by altering the distribution of ground currents^32,33^. Nevertheless, all the CPWs consistently display high internal quality factors above 1000.

### Impact of magnetic field

For future qubit applications, information on the response of the CPWs to magnetic fields will be important. We study the in-plane characteristics using sample B, which contains a CPW with *f*_*r*_ = 3.76 GHz and ${Q}_{i,{B}_{\parallel }=0}=1150$ measured at base temperature, high photon number and zero applied field. Note that vortices in the magnet coil likely decrease the quality factor from its true zero-field value. The design of the transmission line is the same as used for sample A, and the coupling length of the same order. The in-plane field, defined as shown in Fig. 3 (b), is produced by a vector magnet, which allows for a correction of a small sample tilt estimated to *θ* ≃ 0.12° by adjusting different axes of the vector magnet during the sweep.

**Fig. 4:**
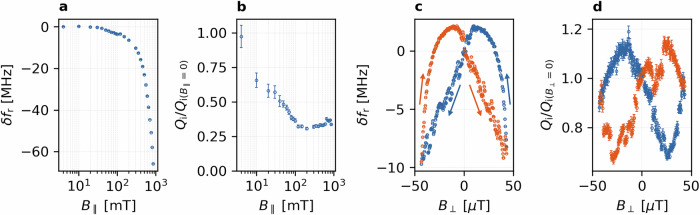
Magnetic field dependence. **a** Shift of the resonance frequency and **b** normalized quality factor as a function of in plane magnetic field from zero to *B*_∥_ = 850 mT. **c** Shift of the resonance frequency and **d** normalized quality factor as a function of out-of plane magnetic field from zero to *B*_⊥_ = 50 *μ*T. The arrows and the color (orange for increasing fields and blue for decreasing fields, respectively) indicate the direction of the sweep.

In Fig. 4, we plot the shift in resonance frequency, *δf*_*r*_ = *f*_*r*_(*B*_∥_ = 0) − *f*_*r*_(*B*_∥_ > 0) and the normalized quality factor *Q*_*i*_(*B*_∥_ > 0)/*Q*_*i*_(*B*_∥_ = 0). The resonance frequency starts to shift significantly only at fields above 100 mT, due to lower density of Cooper pairs increasing the kinetic inductance. Regarding the quality factor, we choose to plot relative values, as the presence of vortices in the vector magnet reduces the zero-field *Q*_*i*_ compared to measurements performed in a magnet-free, shielded setup. The quality factor tends to decrease with increasing field, though its behavior is heavily affected by the fluctuations of the vector magnet and the vortices trapped in the superconducting film. Nevertheless, the quality factor has decreased by less than 75% at 850 mT. At this field, we can still perform a reliable fit to the dip in the transmission amplitude. The large in-plane field resilience is another hint toward an Al film that is thinner than expected.

The CPW’s behavior in out-of-plane magnetic fields is studied using sample C, which carries a CPW with *f*_*r*_ = 3.45 GHz and ${Q}_{i,{B}_{\perp }=0}\simeq 5000$. The results are shown in Fig. 4(c, d), showing a pronounced hysteresis. While the best possible quality factors are likely obtained by releasing vortices after each change of the magnetic field, such a scenario is not realistic for actual (qubit) device measurements. Hence, it is important to analyze the hysteresis, which we believe is caused by an interplay between vortex dynamics in the superconducting film and quasiparticle excitation and trapping. The out-of plane field is supplied by a homemade superconducting coil mounted on the back of the PCB; all the components are encapsulated in an Al magnetic field shield. Starting from zero field, the resonance frequency decreases gradually with increasing field. This is partly due to a narrowing of the effective width of the superconducting region in the center line, and partly due to a slow buildup of vortices^34,35^. Superimposed on this trend, we observe noise attributed to flux avalanches^36^, clearly discernible in Fig. 4(c) as jumps in the *f*_*r*_ dependence as the field is swept from zero towards positive/negative values. When sweeping the field back towards zero, vortices are rapidly annihilated^34,35^ by antivortices formed due to the local internal field reversing polarity before the external field crosses zero^36^. The decreasing vortex density causes a sharp rise in resonance frequency, which reaches a maximum just before the applied field returns to zero, indicating a point of minimal vortex density.

Concurrently, the quality factor initially increases with field due to trapping of quasiparticles by vortices. However, as the vortex density grows, their dissipative effect outweighs the benefits of quasiparticle trapping, and the quality factor begins to decline. Upon sweeping the field back to zero, the quality factor drops even further, although the strong frequency increase suggests efficient vortex annihilation. We interpret this somewhat uncommon observation as an indication that vortices in our system are highly effective at suppressing quasiparticle-induced loss. Their rapid removal, therefore, enhances the effect of quasiparticle dissipation. The quality factor starts to rise at small fields, when the quasiparticle density decreases and their trapping becomes more efficient.

## Discussion

We demonstrate high-quality CPWs in a Ge-based hybrid HS. Unlike previous implementations of such CPWs, our Ge/SiGe QW structure is grown on a Ge substrate, and the Al film is deposited in-situ, via MBE, as an integral part of the hybrid HS. This approach enables to grow a compact semiconductor part of the hybrid HS without introducing a lossy Ge-on-Si interface. The in-situ MBE growth also facilitates the realization of ultra-thin Al films with a high kinetic inductance. While this is not the focus of this study, we suspect the in-situ integration of the hybrid HS to additionally provide a clean superconductor-semiconductor interface (see TEM in Supplementary Fig. 1).

We extract internal quality factors, with *Q*_*i*_ ≈ 49,000 in the quantum regime (single-photon), *Q*_*i*_ > 20,000 in the unsaturated TLS regime, and we measure *Q*_*i*_ up to *Q*_*i*_ ≈ 60,000 at high power. These values suggest long coherence times even when integrated with a quantum device. The CPWs are compatible with large in-plane magnetic fields corresponding to Zeeman energies of 10 μeV for typical in-plane *g*-factors in Ge quantum dots^26,37^. We also characterize the hysteresis in resonance frequency and quality factor under out-of-plane magnetic fields, indicating a complex interplay between quasiparticles and vortices. Comparing our results with previous works, we presume that the absence of the defects caused by the Ge-Si interface enables larger quality factors, while the thin MBE-grown Al film facilitates a large superconducting gap and increased magnetic field resilience.

## Methods

### Heterostructure growth

The hybrid superconductor/semiconductor HS is MBE-grown with nominally 8 nm thin Al film directly grown on top of the Ge/SiGe HS stack (see Supplementary Fig. 1).

### Fabrication

The TL and the CPWs are carved out of the Al film by standard Transene-D wet-etching of the Al followed by reactive ion etching (RIE) of the HS. The Ge/SiGe QW remains intact below the CPW.

### Setup

Unless specified otherwise, the experiments are carried out in a DR with a base temperature of 10 mK, using a Rohde&Schwarz ZNB8 vector network analyzer. The input line is attenuated inside the DR, allowing for low powers down to below one average circulating photon in the CPW. We measure the transmitted signal through the TL *S*_21_, which travels through a superconducting coaxial cable up to 4 K, where it is amplified by a Low Noise Factory LNF_LNC1_12A High Electron Mobility Transistor (HEMT) amplifier, the signal is amplified again at room temperature with an additional amplifier Miteq AMF-3F-01000400-08-10P.

## Supplementary information

Supplementary Information

## Data Availability

The data that support the findings of this study are openly available in ZENODO at https://zenodo.org/records/18229871.
