# Supplementary material for: High-quality and field resilient microwave resonators on Ge quantum well heterostructures
Source: npj Quantum Inf. 2026 Jun 11;12(1):118. doi: 10.1038/s41534-026-01297-w (PMC13407160; doi:10.1038/s41534-026-01297-w)
Supplement: Supplementary file 1 — Supplementary Information [file 41534_2026_1297_MOESM1_ESM.pdf]

Supplementary Information

Luigi Ruggiero[1], Carlo Ciaccia[1], Pauline Drexler[2], Vera Jo Weibel[1], Christian Olsen[1], Christian Schönenberger[3, 4], Dominique Bougeard[2], and Andrea Hofmann[1, 3]

[1] University of Basel, Klingelbergstrasse 82, 4056 Basel, Switzerland
[2] University of Regensburg, Universitaetsstraße 31, 93053 Regensburg, Germany
[3] Swiss Nanoscience Institute, Klingelbergstrasse 82, 4056 Basel, Switzerland
[4] YQuantum, Parkstrasse 1, 5234 Villigen, Switzerland
June 8, 2026

Supplementary Note 1

The transmission line (TL) is designed to be 50 Ω-matched, however considering the relation $L_{\mathrm{kin}} >> L_{\mathrm{geo}}$ valid for our superconducting film, this is not trivial and straightforward. A first resonator couple to a TL was designed in order to estimate L_{kin}, afterwards this value was used as input in *Sonnet* to estimate the optimal dimensions of the TL in order to achieve the optimal 50 Ω. The value is based on what is extracted for the simulation, however, the transmission amplitude in our experiments matches well the amplification and attenuation of the lines, proving the TL impedance is not too far off 50 Ω.

Supplementary Figure 1

The heterostructure is grown on a 475 µm thick commercial germanium (Ge)(001) substrate using solid-source molecular beam epitaxy (MBE). To gradually relax the strain created by lattice mismatch, a 670 nm concentration graded $Si_{1-x}Ge_x$ buffer using the concept of linear grading of x is deposited onto the substrate. The final composition is $Si_{0.2}Ge_{0.8}$. The graded buffer is followed by a $Si_{0.2}Ge_{0.8}$/Ge/$Si_{0.05}Ge_{0.95}$ quantum well (QW), with the respective layer thicknesses 460/15/5 nm.
The superconductor/semiconductor (super/semi) hybrid heterostructure is then completed in an MBE chamber dedicated to metal and oxide growth. The transfer between the chambers is performed without breaking the ultra-high vacuum (UHV, $<10^{-10}$ mbar). To favor the growth of a continuous, crystalline 8 nm aluminium (Al) film, the substrate is cooled to approximately $-100°$C before the Al growth. To prevent uncontrolled oxidation of the Al layer in air after the epitaxy, a 2 nm aluminum oxide capping layer is deposited in situ. Subsequently, the wafer is transferred, again without breaking the UHV, into a dedicated oxidation chamber, where it is exposed to pure oxygen at $3{\cdot}10^{-6}$ mbar for 10 minutes.
Supplementary Fig. 1 shows a high-angle annular dark-field (HAADF) transmission electron micrograph (TEM) of the as-grown interface between the $Si_{0.2}Ge_{0.8}$/Ge/$Si_{0.05}Ge_{0.95}$ semiconductor QW and the Al superconductor film.

Supplementary Figure 2

Supplementary Figure 3

Supplementary Figure 4

Supplementary References

[1] W. C. Dunlap and R. L. Watters, Physical Review **92**, 1396 (1953), ISSN 0031-899X, URL https://link.aps.org/doi/10.1103/PhysRev.92.1396.

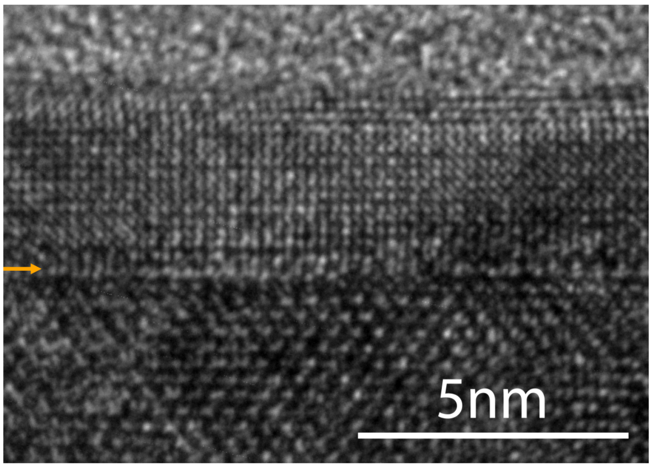

Supplementary Fig. 1: **TEM characterization**. HAADF image of the super/semi interface indicated by the orange arrow, with on top the Al and on the bottom the $Si_{0.05}Ge_{0.95}$ top barrier of the QW.

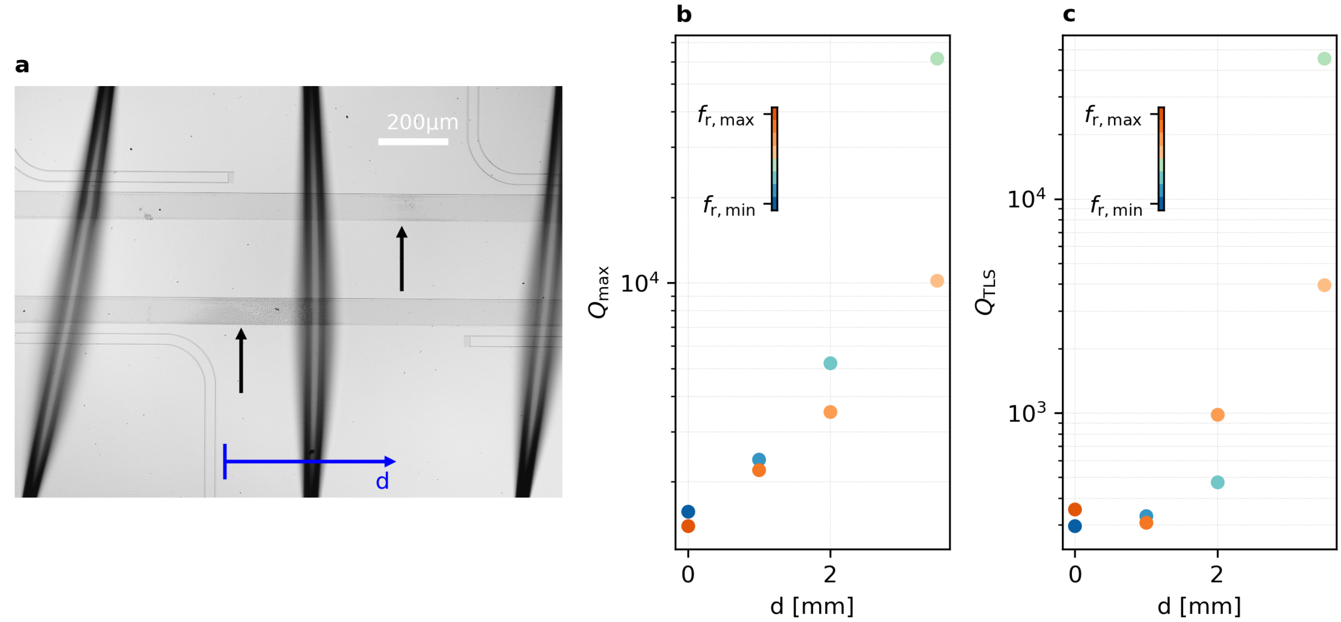

Supplementary Fig. 2: **Impact of inhomogeneous etching on the** Q_i. (a) Optical image of the sample with focus on the inhomogeneous etching of the TL trenches indicated by the black arrows. (b) Max internal quality factor as a function of the distance d from the "dirty spot" in (a). (c) Q_{TLS} defined as the difference between the Q_i at high photon number and saturated TLS and the Q_i at low photon number as a function of the distance d from the "dirty spot" in (a).

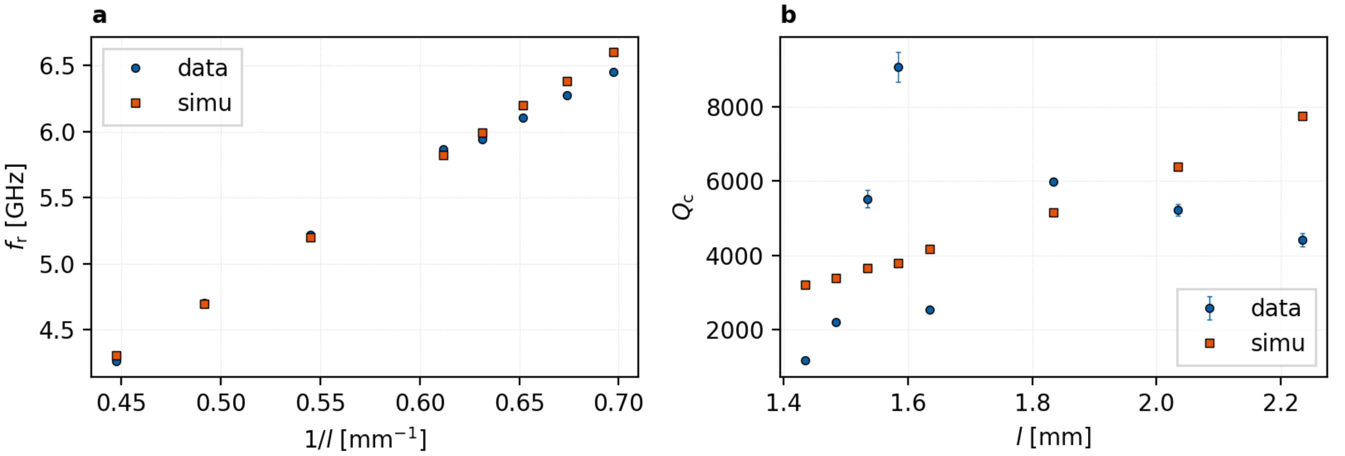

Supplementary Fig. 3: **Sonnet simulations**. (a) Comparison between f_r as a function of length extracted from the data and estimated from the simulations using $L_{kin} = 36\,\mathrm{pH}/\square$ and ε_{rel} of bulk Ge of 15.8 [1] as input parameters. (b) Comparison between Q_c as a function of length extracted from the data as a function of length and estimated from the simulations. Except for some fluctuations the data are in agreement with the theory.

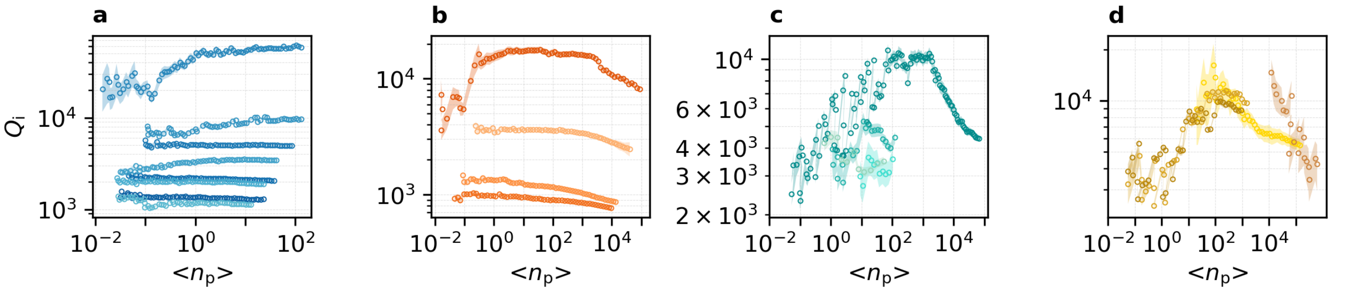

Supplementary Fig. 4: **Power dependence for multiple samples.** (a) Sample A in this manuscript. (b) Sample with four CPWs measured in similar conditions as for sample A. (c) Sample with one CPW measured in a setup with incomplete magnetic field shielding. No bridge bonds used. (d) Same as for the sample in (c).
